# Nanoscopic and Macro-Porous Carbon Nano-foam Electrodes with Improved Mass Transport for Vanadium Redox Flow Batteries

**DOI:** 10.1038/s41598-019-53491-w

**Published:** 2019-11-27

**Authors:** Ibrahim Mustafa, Rahmat Susantyoko, Chieh-Han Wu, Fatima Ahmed, Raed Hashaikeh, Faisal Almarzooqi, Saif Almheiri

**Affiliations:** 1 Department of Chemical Engineering, Khalifa University of Science and Technology, Masdar Institute, Masdar City, P.O. Box 54224, Abu Dhabi, United Arab Emirates; 2 Research & Development Center, Dubai Electricity and Water Authority (DEWA), Dubai, United Arab Emirates; 3 Department of Mechanical Engineering, Khalifa University of Science and Technology, Masdar Institute, Masdar City, P.O. Box 54224, Abu Dhabi, United Arab Emirates; 4 Engineering Division, New York University Abu Dhabi, Abu Dhabi, United Arab Emirates

**Keywords:** Batteries, Batteries, Carbon nanotubes and fullerenes

## Abstract

Although free-standing sheets of multiwalled carbon nanotubes (MWCNT) can provide interesting electrochemical and physical properties as electrodes for redox flow batteries, the full potential of this class of materials has not been accessible as of yet. The conventional fabrication methods produce sheets with micro-porous and meso-porous structures, which significantly resist mass transport of the electrolyte during high-current flow-cell operation. Herein, we developed a method to fabricate high performance macro-porous carbon nano-foam free standing sheets (Puffy Fibers, PF), by implementing a freeze-drying step into our low cost and scalable surface-engineered tape-casting (SETC) fabrication method, and we show the improvement in the performance attained as compared with a MWCNT sheet lacking any macro pores (Tape-cast, TC). We attribute the higher performance attained by our in-lab fabricated PF papers to the presence of macro pores which provided channels that acted as pathways for electrolytic transport within the bulk of the electrode. Moreover, we propose an electrolytic transport mechanism to relate ion diffusivity to different pore sizes to explain the different modes of charge transfer in the negative and the positive electrolytes. Overall, the PF papers had a high wettability, high porosity, and a large surface area, resulting in improved electrochemical and flow-cell performances.

## Introduction

Vanadium redox flow batteries (VRFBs), first proposed by Skyllas-Kazacos *et al*.^1,2^, have received much attention for research and development over the past decades, and currently achieve high energy densities (20–35 Wh L^−1^)^3,4^ and cell peak power densities (≤1340 mW cm^−2^)^5–7^, low crossover rates with regenerative capacities^8^, and high energy efficiencies (>80%)^9–12^. VRFBs consist of stacks made up of cells (membrane, electrodes, gaskets, flow plates, current collectors and fittings) connected in series or parallel, along with a set of pumps, controllers, and external tanks (containing vanadium >1.7 M in aqueous sulfuric acid or hydrochloric acid based solutions). The basic electrochemical operation of the cells are based on the following half-cell redox reactions (Eqs 1, 2 and 3)^13^:

1 $${{{\rm{VO}}}_{2}}^{+}+\,2{{\rm{H}}}^{+}+{{\rm{e}}}^{-}\leftrightarrow {{\rm{VO}}}^{2+}+{{\rm{H}}}_{2}{\rm{O}}\,\,\,\,\,{E}^{\circ }=0.80\,{\rm{V}}\,{\rm{vs}}\,{\rm{Ag}}/{\rm{AgCl}}$$

2 $${{\rm{V}}}^{2+}\leftrightarrow {{\rm{V}}}^{3+}+{{\rm{e}}}^{-}\,\,\,\,\,{E}^{\circ }=-\,0.46\,{\rm{V}}\,{\rm{vs}}\,{\rm{Ag}}/{\rm{AgCl}}$$

3 $${{{\rm{VO}}}_{2}}^{+}+2{{\rm{H}}}^{+}+{{\rm{V}}}^{2+}\leftrightarrow {{\rm{VO}}}^{2+}+{{\rm{H}}}_{2}{\rm{O}}+{{\rm{V}}}^{3+}\,\,\,\,\,{E}_{{\rm{cell}}}^{\circ }=1.26\,{\rm{V}}\,{\rm{vs}}\,{\rm{Ag}}/{\rm{AgCl}}$$

Several studies have been performed to investigate the different forms of carbon as electrodes for VRFBs. Having different chemical and physical properties, a large range of activities toward the VO^2+^/VO_2_^+^ and V^2+^/V^3+^ redox couples were reported, with standard heterogeneous rate constants (*k*°) ranging from 10^−7^ to 10^−3^ cm s^−1 ^^14–22^. Several treatments have been reported in literature to increase the activity of carbonaceous electrodes in VRFBs; For instance, the formation of oxygen containing surface functional groups through electrochemical^23–25^, thermal^26,27^, chemical^28,29^, and acid^30^ treatments have demonstrated improvements in the activity of the electrodes in terms of peak currents and reversibility. Likewise, metal and metal oxides modifications including Ir^31^, Bi^9^, CeO_2_^32^, ZrO_2_^33,34^, PbO_2_^10^, Mn_3_O_4_^35,36^, also demonstrated enhanced activities due to the increased numbers of active sites.

Recently, particular interests in carbonaceous nanomaterials have been growing due to their abilities to facilitate large surface areas, good wettability, and high electrical conductivities^37–41^; Investigations included the deposition of graphene oxides^42,43^, graphene nano-platelets^44^, nitrogen-functionalized nanospheres^45^, MWCNT^46,47^, carbon nanosheets^48^, and carbon nanofibers^49^ on carbonaceous electrodes (CF, CP, GF and GC). Metal and metal oxides/nanomaterials additives on carbon based substrates including Pt/MWCNT^50^ and Mn_3_O_4_/MWCNT^51,52^ have also been demonstrated to improve the electrodes’ kinetics.

With regards to using free-standing sheets of MWCNT as electrodes for redox flow batteries, our previous works^53–55^ demonstrated that these electrodes exhibit enhanced electrochemical and physical properties. Moreover, our in-lab fabricated MWCNT sheets showed high electrochemical activities toward the VO^2+^/VO_2_^+^ and V^2+^/V^3+^ redox couples as compared with the literature^53^. However, our in-lab fabricated MWCNT sheets faced a number of challenges; one of which is that although high electrochemical performance was demonstrated in our cyclic voltammetry and electrochemical impedance spectroscopy (EIS) tests (electrochemical tests that employ low currents procedures), low performance (energy efficiencies <70%) was achieved in our charge-discharge tests (flow battery setup tests that employ high current procedures). The discrepancy in performance between low current and high current operations made us hypothesize that mass transport plays a critical role. We suggest that electrolytic mass transport may have been hindered due to meso-porous properties of our first generation MWCNT sheets (the sheets had tiny pores (<100 nm) which may have slowed down electrolyte transport across the bulk of the electrode). Second, the conventional MWCNT sheets fabrication methods (vacuum filtration) that we utilized during our previous studies were energy intensive, making them costly and limiting their applicability for commercialization. Finally, the as-fabricated MWCNT sheet size was limited to the size of the filtration membrane, making them non-scalable, which poses a great challenge for the effective development and commercialization of the electrodes. Thus, we were motivated by these important issues to investigate new scalable methods to fabricate free-standing MWCNT sheets that can facilitate better mass transport in redox flow batteries.

Several methods to develop macro-porous carbonaceous foams from nanoscopic CNTs have been devised in the literature; following the Shaffer and Windle method^56^ in which the poly(vinyl achohol) gas-liquid phase separation (foaming) process was first suggested, several researchers demonstrated the preparation of foams from MWCNT composites with controllable pore structure including ethylene vinyl acetate copolymer/MWCNT^57^, polyurethane foam/MWCNT^58^, poly(methyl methacrylate)/MWCNT^59,60^, and styrene-divinylbenzene-based high internal phase emulsions/MWCNT^61^. Other studies demonstrated the applicability of a solid-liquid phase separation method to produce CNT based foams and included SWCNTs within a gelatin gel template through freeze drying and subsequent thermal heating^62^, chitosan/MWCNT composite by freeze drying of a 1 wt % chitosan acetic acid aqueous solution doped with MWCNT^63^, carboxymethyl cellulose (CMC) sodium salt solution/MWCNT through freeze-drying at different process conditions which resulted in smaller macro-porosity when a faster cooling rate, a higher surfactant concentration, or a higher MWCNT content was employed^64^, non-aligned meso- and macro-porous MWCNT cyrogels through gelation, flash freezing in liquid nitrogen, and subsequent freeze drying, as well as aligned MWCNT cyrogels though ice templating and subsequent freeze-drying of silk fibroin/MWCNT^65^, non-aligned chitosan/MWCNT composites by freezing in liquid nitrogen and subsequent freeze-drying, which allowed the formation of regular and irregular monoliths of different shapes, all of which demonstrated the possibility of fabricating macro-porous structures based on CNTs.

With regards to the application of macro-porous CNT/foam-based materials in electrochemical devices, several experiments were reported; Gutierrez *et al*. demonstrated improvements in the attainable current density (up to 242 mA cm^−2^) when Pt decorated chitosan/MWCNT were employed as an anode material in direct methanol fuel cells, which was attributed to their 3D inter-connected macro-porous structure offering efficient reactant and product diffusion, and lower levels of poisoning^66^. Dong *et al*. illustrated that free-standing graphene-CNT hybrid foam electrodes can be promising for electrochemical sensing applications to their large active surface area and rapid charge transfer processes facilitated by its 3D macro-porous foam structure^67^. Yuan *et al*. demonstrated high performance of macro-porous CNT-S paper with high sulfur loading in lithium-sulfur batteries, having an initial discharge capacity of 6.2 mAh cm^−2^. Chervin *et al*. demonstrated the applicability of carbon nanofoam-based cathodes for lithium-oxygen batteries with a cathode specific capacity of 1000–1250 mAh g^−1^ at 0.1 mA cm^−2^, which was twice of that illustrated by the meso-porous nanofoams (580–670 mAh g^−1^), and suggested that the interior pore volume may be underutilized when the electrode thickness is increased (180–530 µm for their case)^68^.

Herein, we utilized a low energy consuming and scalable MWCNT fabrication method, as described in our previous works^69,70^, to fabricate meso-porous tape casted free-standing MWCNT sheets (TC). We also modified the process to incorporate a freeze drying step followed by a compression step to attain a larger range of pore sizes in the tape casted free-standing carbon nano-foam sheets (PF). Our method sets important basis to the advancement of scalable and macro-porous MWCNT electrodes which can be promising for use in redox flow batteries.

## Experimental

### Chemicals

To prepare the positive electrolyte, vanadium(IV) oxide sulfate powder (VOSO_4_, 97%, Changsha Asian Light Economic Trade Co., Ltd, China) was dissolved in a 3 M solution of aqueous sulfuric acid (H_2_SO_4_, 96.2%, Sigma-Aldrich, USA) utilizing deionized (DI) water (resistivity >18.0 MΩ × cm, Purite Select Fusion water purification system, Thame, UK). The negative electrolyte (V^3+^) was electrochemically generated by charging an H-cell at a constant voltage of 1.5 V until a cutoff value of less than 10 mA was attained.

### Fabrication of electrodes

**Figure 1:**
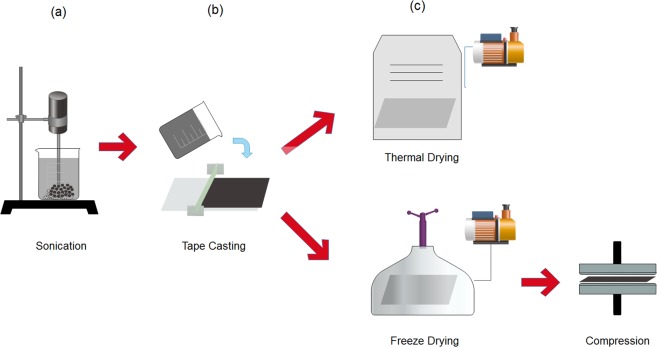
Scalable and low energy consuming method for the fabrication of freestanding sheets of MWCNT with variable pore sizes; (**a**) a suspension of MWCNT in 15% ethanol solution was sonicated, (**b**) the sonicated suspension was tape casted on a copper substrate, and (**c**) the casted MWCNT layers were dried overnight using a vacuum oven at 80 °C to achieve a micro and a meso-pore sized distribution of MWCNT, or freeze dried and subsequently compressed to create macro pores within the pore size distribution of the MWCNT sheets.

MWCNT flakes (Applied Nanostructured Solutions, L.L.C., USA) produced by a chemical vapor deposition process with an average length of 30 µm, were utilized in this study^71^. The MWCNT flakes consisted of 3% polyethelyne glycol (PEG) which acted as a surfactant to enhance dispersion, and increase hydrophilicity. Meso-porous freestanding sheets (Tape-cast, TC) were fabricated in the form of a buckypaper, using a surface engineered tape casting (SETC) method as described in our work^69^; 0.4 g of MWCNT were dispersed in a solution of 200 mL (15% ethanol), and the resulting suspension was sonicated for 10 min at a power of 40 W. The dispersion was then gently poured over a copper sheet and tape casted to achieve 5 mm of thickness of the wet casted layer. The layers were then dried in a vacuum oven at 80 °C for 12 h. To fabricate the macro-porous freestanding MWCNT sheets (Puffy Fibers, PF), the same process has been followed except for the last step; instead, the wet casted layer was transferred to a conventional freezer operating at −20 °C for 12 h to achieve complete freezing. The frozen casted layer was then transferred to a 4.5 L freeze drier (Labonco Co., USA) under 10 microHg vacuum for 24 h, to reduce the pressure at P < P_triple point_ to achieve sublimation of the frozen solvent. To increase its mechanical strength whilst maintaining its puffiness, the dried sample was then placed between 2 platens and compressed at room temperature, under a clamping force of 500 kg for 1 min. The fabrication process of both electrodes is shown graphically in Fig. 1.

### Physical and chemical characterization

Morphological characterizations of the electrodes were performed using a scanning electron microscope (SEM, Quanta 250, FEI, Oregon, USA) under high vacuum at moderate magnifications, and condensation experiments were performed using the environmental scanning electron microscope module (E-SEM) using the same device at a pressure of 700 Pa, and a stage cooling temperature of 1 °C. Transmission electron microscope (Tecnai T20, 200 kV, FEI, USA) was employed at higher magnifications. The macro-porosity analysis was performed by probing images in a fixed area of 250 × 250 µm using SEM, in which the numbers and radii of macro-pores were counted and measured. The meso-porosity analysis was performed through physisorption of nitrogen gas at 77 K with a surface area analyzer (Quantachrome Nova 2000e). The samples were first degassed at 150 °C for 5 h, and then the surface area was estimated by means of the multipoint Brunauer–Emmett–Teller (BET) method for values acquired over the linear region (*P*/*P*_0_ = 0–0.35) of the desorption isotherm. The relative density and porosity were calculated using a gas pycnometer (Accupyc 1340TC, Micromeritics Instrument Co., USA). Defects were characterized by using Raman Spectroscopy at a laser excitation of 532 nm (alpha 300 RAS, WITec GmbH, Ulm Germany). Surface functionality was characterized using Fourier transform infrared (FTIR) spectrometer (VERTEX 80 V, Bruker Optik GmbH, Germany), by plotting transmittance plots for sample pellets consisting of a mixture of the sample and KBr powder in a 1:180 mass ratio. Wettability was investigated by using a goniometer (DM-501, Kyowa Interface Science, Japan) with a droplet volume of 20 μL. Finally, a four-probe hall-effect measurement system (7607, Lakeshore, USA) was used to measure the electrical conductivity, and silver paste was utilized on the 4 corners of each of the 2 × 2 cm samples to reduce the effect of contact resistances between the measurement pins and the sample.

### Electrochemical characterization

Cyclic voltammetry and electrochemical impedance spectroscopy were performed in a three-electrode-cell configuration, utilizing a sheet of the investigated electrode (TC or PF) as a working electrode, along with a Ag/AgCl reference electrode and a platinum sheet counter electrode. Measurements were performed using an Autolab PGSTAT302N potentiostat (Metrohm Autolab B.V., Netherlands). The positive and the negative electrolytes were prepared using 0.05 M VOSO_4_ in 3 M H_2_SO_4_ and 0.05 M V^3+^ in 3 M H_2_SO_4_ aqueous solutions respectively. The cyclic voltammetry methods employed were based on a potentiostatic staircase method over the respectively investigated potential range, and were compensated for iR-drop by using an electrochemical impedance spectroscopy (EIS) method. The EIS results were acquired at the formal potentials with frequencies ranging between 10 kHz and 0.1 Hz and at an amplitude of 10 mV RMS. To control the working area of the investigated MWCNT sheets, molten wax was applied over the non-working areas to achieve a working area of 0.1 cm^2^ for all 3-electrode cell experiments.

For flow-cell performance investigations, charge–discharge experiments were performed in a 16 cm^2^ flow cell (Fuel Cell Technologies Inc., USA). Each half-cell of the setup consisted of a graphite flow field plate (Poco® Graphite) with single serpentine flow pattern, a gold-plated copper current collector, a Teflon® gasket (McMaster-Carr, USA) for sealing, 1 stainless end plate, and a single layer of our in-lab fabricated MWCNT sheet under investigation. A single layer of Nafion® N117 membrane (DuPont, USA) was employed between the positive and the negative half-cells and was obtained from Ion Power Inc. (USA). The membrane was pretreated by soaking in a 1 M aqueous sulfuric acid solution at 80 °C for 2 h and subsequent rinsing with deionized water. The electrolyte consisted of 1.7 M VOSO_4_ in 3 M H_2_SO_4_ solution. 90 mL of electrolyte volume was used for each half-cell, and was pumped at room temperature by means of peristaltic pumps (Masterflex) at a fixed flow rate of 30 mL min^−1^. First, V^3+^ and VO_2_^+^ electrolyte states were generated by charging at a constant voltage of 1.5 V until a current of less than 10 mA was reached. Next, the generated VO_2_^+^ electrolyte was removed from the positive half-cell and was replaced with a fresh solution of VO^2+^, such that the system consisted of 90 mL V^3+^ negative electrolyte and 90 mL VO^2+^ positive electrolyte (A typical discharged stated of a VRFB). Charge–discharge was then performed at a constant current of 50 mA cm^−2^ with cutoff voltages of 1.7 and 0.8 V. Throughout operation, the electrolyte containers were well sealed and high-purity N_2_ gas was bubbled into the negative electrolyte to prevent unwanted chemical oxidation by air.

## Results and Discussion

### Morphological and physical characterization of electrodes

#### Microscopy and porosity analysis

**Figure 2:**
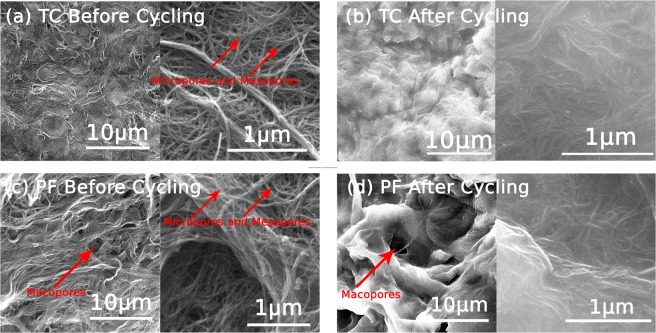
SEM images of TC electrodes prepared via the thermal drying route obtained (**a**) before cycling and (**b**) after cycling, and SEM images of PF electrodes prepared via the freeze-drying route obtained (**c**) before cycling and (**d**) after cycling. The images show that larger pore sizes are attainable for the PF electrode as compared with the TC electrode, which sustained their macro-form over cycling.

First, the morphology of our in-lab fabricated electrodes were probed and compared using SEM at various magnifications. As can be seen in Fig. 2a,b, both electrodes consisted of entangled MWCNT fibers (Diameters = 10 to 13 nm, See Supporting Information, Fig. S1), but were more closely packed in the TC electrode as compared with PF electrode. Moreover, the three-dimensional arrangement of the MWCNT structure was porous in both electrodes; however, it consisted of larger pore sizes for the PF electrode as compared with the TC electrode. The TC electrode had a smaller thickness (20 µm), a smaller porosity (0.71), and a higher electrical conductivity (7.38 × 10^3^ S m^−1^) as compared with the PF electrode (150 µm, 0.85, and 6.81 × 10^3^ S m^−1^ respectively). This implies the advantages that the PF electrode possesses over the TC electrode because larger porosity can facilitate improved mass transport of the electrolyte during cycling operation, which is a very important parameter that can reduce concentration polarization losses. Nevertheless, the PF electrode exhibits a lower electrical conductivity as compared to the TC electrode. SEM images probed after cycling (Fig. 2b,d) confirm that both electrodes maintained a good morphological structure which remained in its entangled nanoscopic fiber form. Moreover, it is notable that the macro-pores in the PF electrode remained opened providing facilitated pathways for diffusion within the electrode’s structure during electrolyte cycling, which was important to improve flow and avoid high pressure losses within the bulk of the electrode. However, the images show that the fibers become more agglomerated after cycling.

#### Porosity and pore size distribution analysis

**Figure 3:**
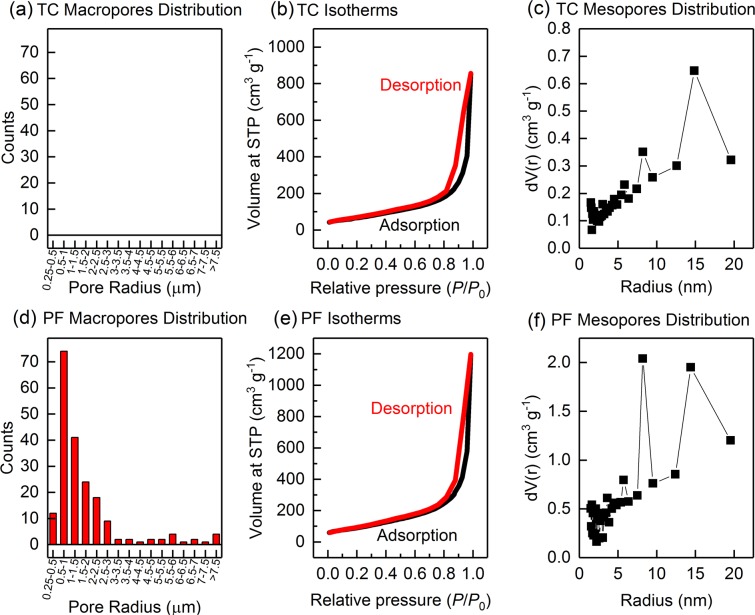
Porosity analysis for the TC electrode showing macro pore distribution obtained through SEM analysis for the (**a**) TC and the (**d**) PF electrodes, Adsorption and Desorption Isotherms obtained through BET for the (**b**) TC and the (**e**) PF electrodes, and meso pore distribution obtained through BJH analysis for the (**c**) TC and (**f**) PF electrodes. The results demonstrate that the PF electrode showed larger BET surface area and larger pore sizes in their morphologies.

**Figure 4:**
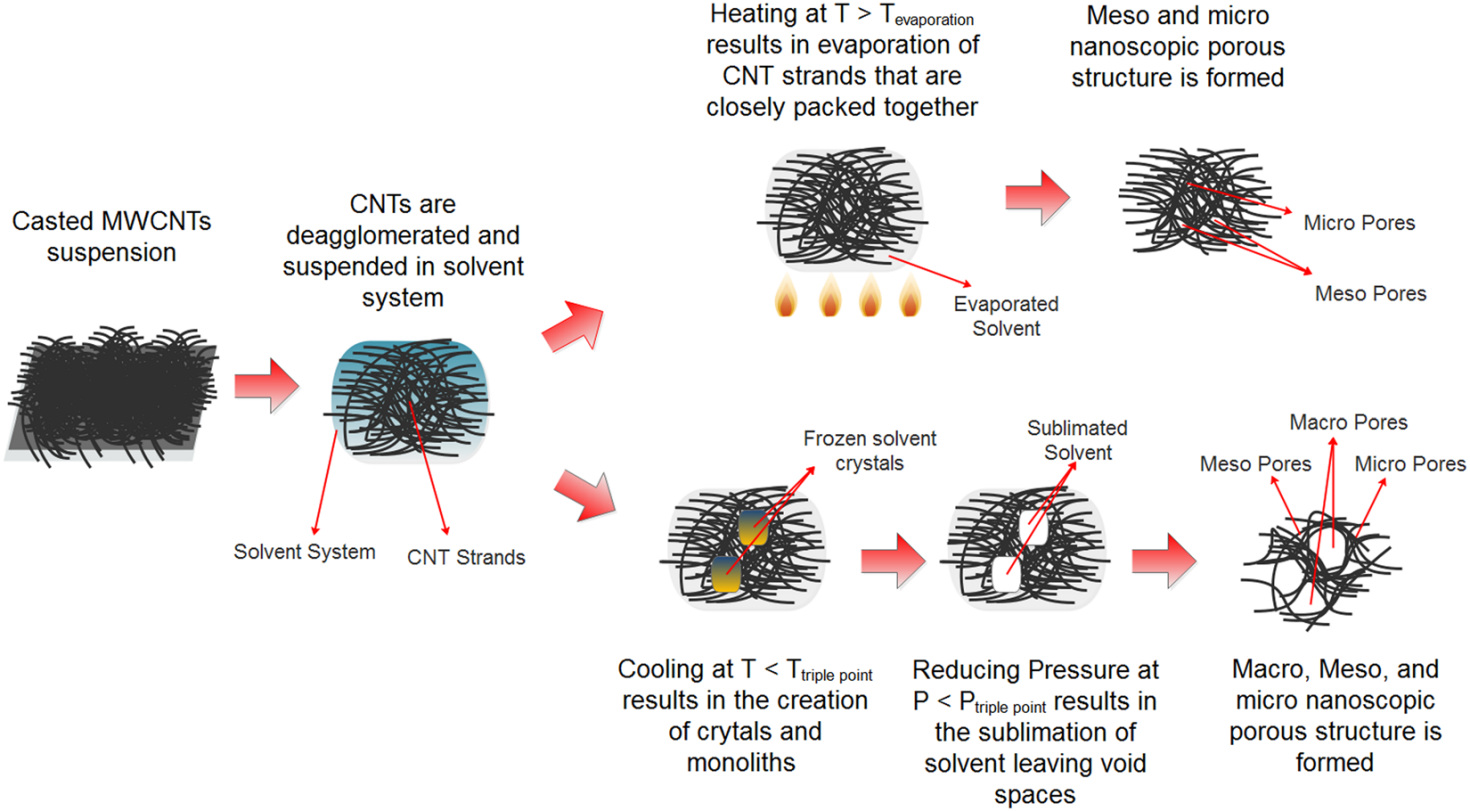
Schematic illustration of the thermal drying and the freezing drying processes utilized to achieve the different pore sized structures TC and the PF electrodes.

To gain further insights into the morphology of our in-lab fabricated electrodes, we performed porosity distribution analysis. First, we counted the number of macro-pores in a fixed area of 250 × 250 µm for each electrode and measured their individual radii using SEM as shown in Fig. 3a,d. The TC electrode did not resemble any macro-pores; whilst the PF electrode resembled a large range of macro-pores distribution with the most recurrent being in the 0.5 to 1 µm range. The larger pores in the PF electrode are attributed to the effect of the slow cooling freezing process, in which macro-sized monoliths of the frozen solvent were crystallized, and which were subsequently sublimated into their vapor form, leaving behind macro-pores. On the other hand, the TC electrode was not frozen, and was dried using thermal heating, which resulted in a finer and smaller pore-sized structure. The mechanism of this process is further elaborated in Fig. 4.

We also performed BET analysis at 77 K using Nitrogen gas and generated the isotherms as shown in Fig. 3b,e. Both electrodes showed hysteresis at the higher P/P_0_ range, indicating meso-porosity of the both electrodes, and the isotherms were analyzed using the multi-point BET method in the linear range (0–0.35 *P*/*P*_0_) of the desorption curves, and showed that the PF electrode had a larger BET surface area (343 m^2^ g^−1^) as compared to the TC electrode (255 m^2^ g^−1^), which can be attributed to the greater isolation or de-bundling of the individual MWCNT fibers in the PF electrode, attributed to the effect of freeze drying. These results are remarkable because they can indicate that a larger electrochemically active surface area may be available for a reaction in the PF electrode^53^. Finally, we investigated the mesoporous distribution of our electrodes by analyzing the adsorption curves using the Barrett-Joyner-Halenda (BJH) method, and the results showed that both electrodes exhibit a mesoporous distribution with the most recurrent size being at 9 nm for the PF electrode and 15 nm for the TC electrode.

#### FTIR analysis

**Figure 5:**
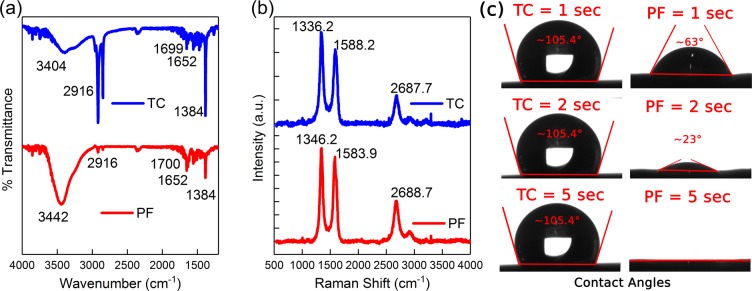
(**a**) FTIR spectra of tape casted electrodes fabricated to achieve different pore size distributions for the TC electrode prepared through a thermal drying route, and the PF electrode prepared through the freeze-drying route. Both electrodes exhibit similar surface chemistry compositions; however, the C-H bond peaks are more pronounced at the TC electrode. (**b**) Raman spectra of the TC and PF electrodes reveal that both electrodes have the three characteristic bands (D, G, and 2D bands), and that the I_D_/I_G_ and I_2D_/I_G_ ratios were higher and lower respectively for the TC electrode as compared to the PF electrode indicating that the number of defects was higher in the TC electrodes. (**c**) Contact angles measured for the TC and the PF samples over time, showing that the PF sample had a higher wettability attributed to its larger pore size and higher porosity creating a wicking effect to absorb fluid within the voids of its structure.

The surface functional groups on the TC and the PF freestanding sheets were investigated through FTIR spectroscopy (Fig. 5a). Transmittance bands at ~3404–3442 cm^−1^ were exhibited by both electrodes and were attributed to the O–H stretching vibrations, related to the effects of sonication in DI water during fabrication of the MWCNT sheets. Bands at ~1384 and ~2916 cm^−1^ were attributed to the C-H bending vibrations which can be attributed to the bending vibration of the alkyl chain of the PEG, and showed a higher intensity in the TC electrode as compared to the PF electrode. Bands at the ~1652 cm^−1^ were attributed to the C=C bond and were resembled by both electrodes, which is attributed to the MWCNT structure. The bands in the 1699–1700 cm^−1^ range were attributed to C=O vibration, and were observed in both electrodes, attributed to carboxyl functional groups existence on both electrodes.

#### Raman spectroscopy

The number of defects in the TC and the PF electrodes were investigated using Raman Spectroscopy (RS) (Fig. 5b). Both electrodes exhibited the three MWCNT characteristic peaks at the 1336–1347 cm^−1^, 1583–1589 cm^−1^, and 2687–2689 cm^−1^ which correspond to the disordered structure peak (D), in-plane C-C bond vibration peak (G), and long-range order peak (2D) respectively. To compare the number of defects in both electrodes, the ratios of the intensities were calculated and compared; the TC electrode showed a higher *I*_D_/*I*_G_ ratio (1.25) and a lower *I*_2D_/*I*_G_ ratio (0.39) as compared with the PF electrode (1.06 and 0.7 respectively), indicating that the TC electrode exhibited a larger number of defects in the MWCNT structure^72,73^. The larger number of defects in the TC sheets may be attributed to the stress inducing effect of thermal drying on the MWCNT strands; As the solvent evaporates, the MWCNT strands shrink together in an uneven manner due to the higher convective heat transfer at the edge regions of the casted sheets, as compared with the relatively less thermally exposed interior sheet regions, and this in turn, resulted in an uneven distribution of localized stresses across the MWCNT strands, increasing the number of defects. On the other hand, the PF sheets were dried through the freeze drying route, which involved sublimation of the solvent directly into its gaseous state, inducing less stresses on the MWCNT strands.

#### Wettability

The wettability of the TC and the PF samples were investigated using goniometry (Fig. 5c). The TC resembled lower wettability toward a 20 µL drop of DI water with contact angles of ~105.4° and maintained that behavior over the testing time of 5 seconds. On the other hand, the PF electrodes demonstrated higher wettability under the same conditions with contact angles of ~63° after 1 second, ~23° after 2 seconds, and no contact angle due to full absorption of the DI water droplet after 5 seconds. Even though the wettability of both electrodes is attributed to their surface performance, the inability of the water droplet to rest on the porous surface played a role in the apparent contact angles resembled by the PF; the large pores acted as channels and prevented the droplet from coming to rest due to a non-zero hydrostatic pressure. This was confirmed by the similar contact angles measured when the macro-porosity effect was excluded by performing ESEM condensation experiments (See Supporting Information, Fig. S2).

### Electrochemical characterization

#### Cyclic voltammetry

**Figure 6:**
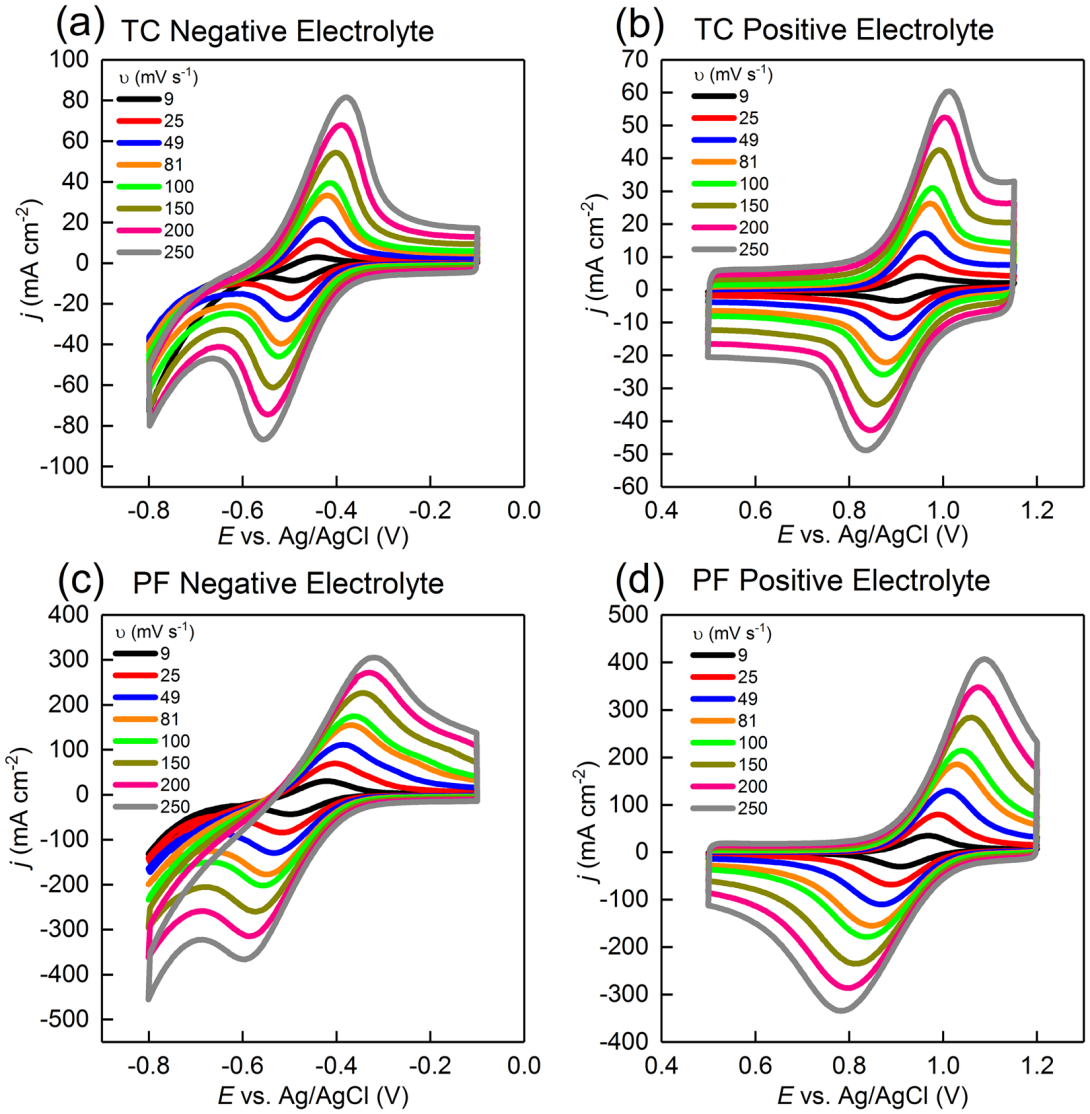
iR-corrected steady-state cyclic voltammograms obtained at different scan rates for the TC electrodes performed at room temperature in (**a**) 0.05 M V^3+^ in 3 M H_2_SO_4_ and (**b**) 0.05 M VO^2+^ in 3 M H_2_SO_4_, and steady-state cyclic voltammograms obtained at different scan rates for the PF electrodes performed at room temperature in (**c**) 0.05 M V^3+^ in 3 M H_2_SO_4_ and (**d**) 0.05 M VO^2+^ in 3 M H_2_SO_4_. Both electrodes were highly active toward the positive and the negative vanadium redox couples. The TC electrode showed lower peak potential separation while the PF electrode showed higher current densities.

Steady state cyclic voltammoragms (CVs) at various scan rates (*v*) were plotted for both the TC and the PF electrodes in a solution of 0.05 M V^3+^ in 3 M H_2_SO_4_ (Fig. 6a,c) and a solution of 0.05 M VO^2+^ in 3 M H_2_SO_4_ (Fig. 6b,d) to investigate their electrochemical activity toward the negative and the positive redox couples of a VRFB. All CVs were reported at the 2^nd^ cycle and showed that both electrodes were electrochemically active toward the VO^2+^/VO_2_^+^ and V^2+^/V^3+^ redox couples.

To gain an in-depth understanding of the electrochemical reversibility of the electrodes, we analyzed the voltammograms shown in Fig. 6. First, we plotted the peak potential separation (Δ*E*_p_) as a function of *v* (Fig. S4a,b in Supporting Information) which ranged from 43.34 to 170.3 mV for the TC electrode and from 59.36 to 298.65 mV for the PF electrode in the positive electrolyte, and from 22.74 to 171.66 mV for the TC electrode and from 69.28 to 274.06 mV for the PF electrode in the negative electrolyte, indicating the irreversibility of the electrodes in both solutions, and the lower Δ*E*_p_ attainable by the TC electrode. Moreover, the ratio of anodic peak current to cathodic peak current (*I*_pa_/*I*_pc_) (Fig. S4c,d in Supporting Information) deviated from unity suggesting that the kinetics at both electrodes were homogenous and/or that there were other complications in the electrode processes. Finally, the peak currents (*j*_p_) were plotted versus scan rate and the results show that the PF electrode facilitated higher peak currents for both the positive and the negative electrolytes which can be attributed to the larger number of active sites available for a reaction in the PF electrode related to its higher BET surface area and improved wettability (as described in Sections 3.1.2 and 3.1.5), as compared with the TC electrode. Moreover, we calculated diffusion coefficients (*D*) and the results (given in Table 1) suggest that the PF electrode facilitated higher diffusion coefficients as compared with the TC electrode. This behavior was consistent for both the negative and the positive electrolytes, and is attributed to the higher wettability and larger pore sizes in the PF electrode.

**Table 1:** Values of diffusion coefficients obtained for the TC and the PF electrodes in the negative and the positive electrolytes.

Electrolyte Electrode	*D*_c_ (cm^2^ s^−1^)	*D*_a_ (cm^2^ s^−1^)
**V**^**3+**^**/V**^**2+**^		
TC	0.35 × 10^−4^	0.19 × 10^−4^
PF	3.19 × 10^−3^	0.41 × 10^−3^
**VO**^**2+**^**/VO**_**2**_^**+**^		
TC	0.12 ×10^−4^	0.24 ×10^−4^
PF	2.37 ×10^−3^	3.94 ×10^−3^

^a^*D*_c_ = cathodic diffusivity; *D*_c_ = anodic diffusivity.

It must be noted that cyclic voltammetry was performed in a stagnant/diffusion controlled solution and may not be sufficient enough to gain full understanding about the kinetics of the reactions, due to the interplay between the lower operational currents and the hindered mass transport within the meso-pores of the electrodes^74,75^, which made it necessary for us to perform further electrochemical investigations.

#### Electrochemical impedance spectroscopy

**Figure 7:**
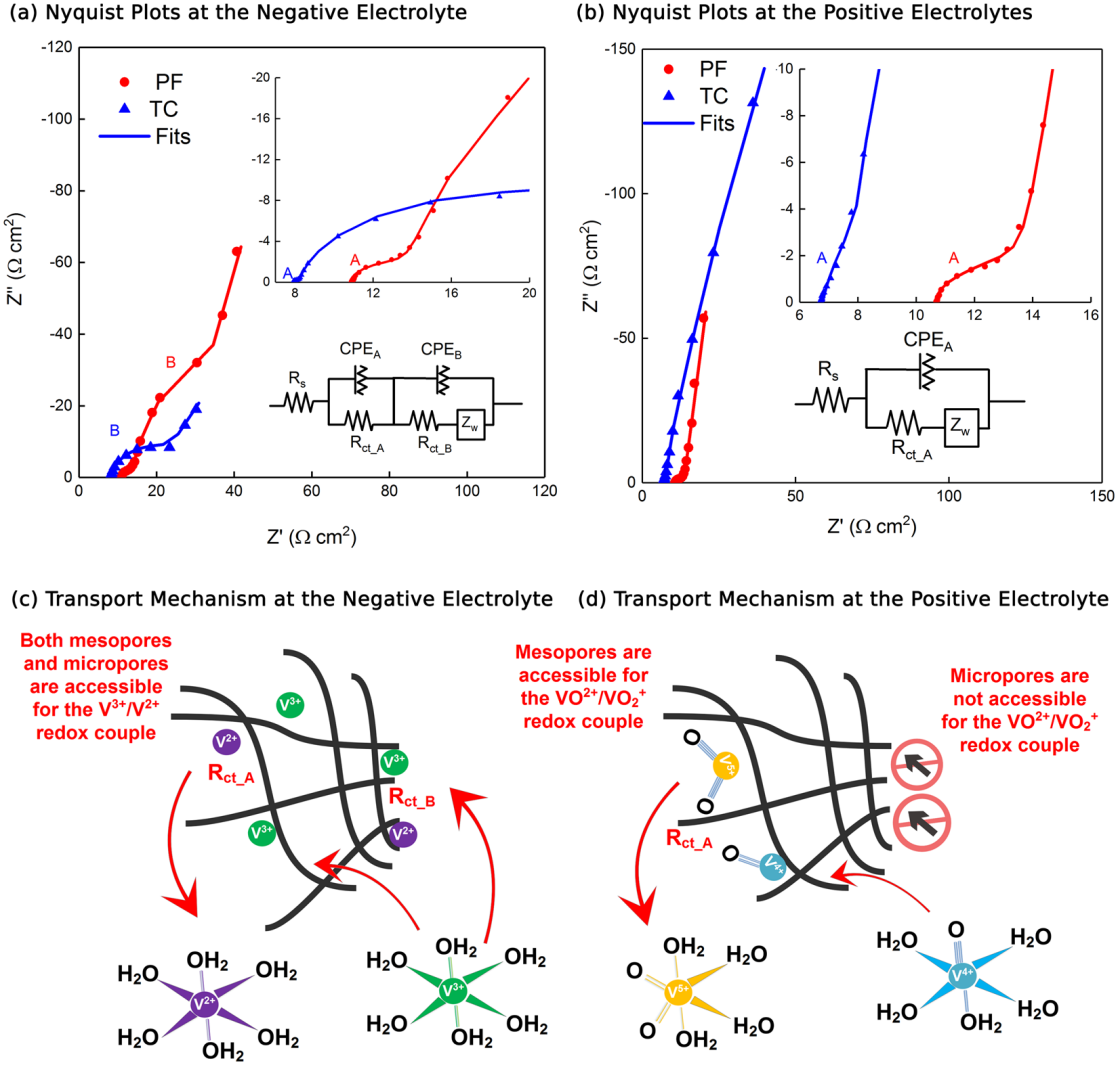
EIS for the TC and the PF electrodes performed in (**a**) the negative and (**b**) the positive electrolytes of a VRFB, indicating that the TC electrodes facilitated lower charge transfer resistance while the PF electrode facilitated higher capacitance. (**c**) Proposed electrolyte transport mechanism of the negative and (**d**) proposed electrolyte transport mechanism of the positive electrolytes within the meso and micro sized pores of the fabricated MWCNT sheets. The V^3+^/V^2+^ redox couples are accessible within both the meso and micro sized pores of the fabricated MWCNT electrodes, resembling 2 charge transfer processes. The VO^2+^/VO_2_^+^ redox couples are less likely to be accessible in the micro sized pores due to their larger ionic radii, giving rise to only 1 charge transfer process within the meso sized pores of the MWCNT electrodes.

We used electrochemical impedance spectroscopy (EIS) to further investigate the kinetics of the TC and PF electrodes for both the negative (V^2+^/V^3+^) and the positive (VO^2+^/VO_2_^+^) vanadium redox couples. This was done by performing EIS in a 3-electrode cell configuration at the formal potentials of the V^2+^/V^3+^ and VO^2+^/VO_2_^+^ redox couples (0.9 V and 0.4 V Vs. Ag/AgCl respectively) in the respective electrolyte. Nyquist plots of both electrodes resembled depressed semi-circles at the high-intermediate frequency regions ascribed to the charge transfer process and resembled a sloped line at the low frequency region ascribed to the Warburg diffusion process. The size of the semi-circles correspond to the charge transfer resistance (R_ct_) of each electrode in the respective electrolyte. The Nyquist plots obtained in the positive electrolyte consisted of only 1 depressed semi-circle (denoted as A in Fig. 7b in the higher frequency regions), whilst those obtained in the negative electrolyte consisted of 2 depressed semi-circles (denoted as A in the higher frequency regions and B in the lower frequency regions in Fig. 7a).

The presence of the 2^nd^ depressed semi-circle for both electrodes in the negative electrolyte can be related to the additional charge transfer process (R_ct_B_) occurring at low frequencies due to the relatively slower diffusion of ions in the micro pores of the MWCNT sheets^76–78^, and can indicate the higher accessibility of the V^3+^/V^2+^ ions in the micro pores. However, this was not the cast in the positive electrolyte. To explain this behavior, we propose the V^3+^/V^2+^ redox couples are accessible within both the meso and micro sized pores of the fabricated MWCNT electrodes, resembling 2 charge transfer processes. On the other hand, the VO^2+^/VO_2_^+^ redox couples are less likely to be accessible in the micro sized pores due to their larger ionic radii, giving rise to only 1 charge transfer process within the meso sized pores of the MWCNT electrodes, as graphically illustrated in Fig. 7c,d. (Recall that both the TC and PF electrodes consisted of a range of variables pore size distributions, as discussed in Section 3.1).

The EIS data were fitted using equivalent circuits (Fig. 7 insets) which utilized a constant phase element (CPE) instead of a pure capacitance element to attain a more accurate fit of the experimental data, and we obtained the charge transfer resistance (*R*_ct_) and the double layer capacitance (*C*_dl_) values to improve our understanding of the electrode’s kinetics and to supplement our CV studies, as given in Table 2.

**Table 2:** Charge transfer resistance (*R*_ct_) and double layer capacitance (*C*_dl_) values obtained by fitting the electrochemical impedance spectra.

Electrode	Electrolyte	${{R}}_{{\bf{ct}}\_{\boldsymbol{A}}}$ (Ω cm^2^)	${{C}}_{{\bf{dl}}\_{\boldsymbol{A}}}$ (µF cm^−2^)	${{R}}_{{\bf{ct}}\_{\boldsymbol{B}}}$ (Ω cm^2^)	${{C}}_{{\bf{dl}}\_{\boldsymbol{B}}}$ (µF cm^−2^)
TC	Negative	0.31	5,827.30	17.60	37,778.80
	Positive	0.49	8697.60	N/A	N/A
PF	Negative	2.84	27,471.60	120.00	209,148.00
	Positive	1.14	17,067.00	N/A	N/A

*R*_ct_ for the TC electrode was consistently lower for both the negative and the positive redox couples indicating the higher activity of the TC electrode as compared to the PF electrode. These results come in accordance with the lower peak separations demonstrated in our cyclic voltammetry analysis findings (as described in Section 3.2.1). On the other hand, *C*_dl_ was consistently higher for the PF electrode, indicating that the amount of active sites available for a reaction is higher for the PF as compared with the TC. These results come in accordance with the higher peak current densities, larger BET surface area, and higher wettability, which were resembled by our PF electrode (as was evident from the CV analysis results shown in Fig. S4 e,f, BET analysis shown in Section 3.1.2 and contact angle measurements shown in Section 3.1.5), as compared with the TC electrode. (Recall that as compared to CV, EIS experiments involve much smaller amplitude potential perturbations - only 10 mV amplitude around the electrode’s formal potential was applied in a diffusion controlled system).

#### Polarization curves

**Figure 8:**
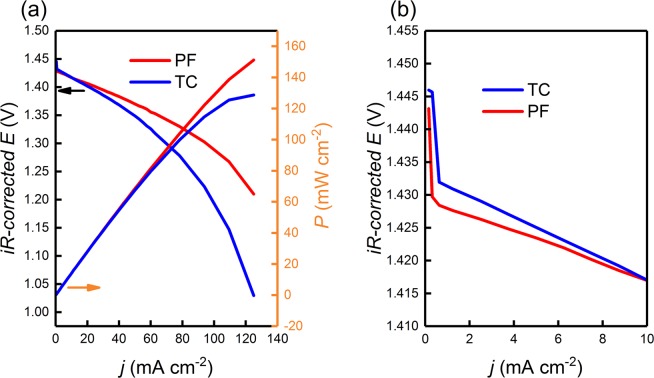
iR-corrected polarization curves for the flow cells incorporating the TC and the PF electrodes, (**a**) at the full applied current density range, showing the ohmic and concentration polarization losses, and (**b**) at low applied current density range magnifying the activation polarization losses region. The plots show that the TC electrode resembled lower activation polarization losses at low current densities, but the PF electrode resembled lower pseudo-iR and concentration losses at higher current density operation.

To gain further insights into the performance of the TC and the PF electrodes, we performed iR-corrected polarization curve studies as described in the literature^79^, at a constant state of charge (SOC = 50%), to investigate the activation, ohmic, and concentration polarization losses associated with each electrode during flowing electrolyte cell operation (Fig. 8). Losses due to activation over-potential are significant when the rate of charge transfer is low and are thus are apparent at low current densities. The flow-cell which incorporated the PF electrode exhibited a higher activation over potential (17 mV) as compared with that of the TC electrode (13 mV). We attribute this behavior at these low rates of charge transfer to the higher reversibility of the TC electrode as compared with the PF electrode (as evident from our cyclic voltammetry results in Section 3.2.1 and EIS analysis in Section 3.2.2). Even though the polarization curves were iR-corrected (compensated only for the ionic and contact resistances), the polarization curves still show the typical behavior caused by ohmic resistance as shown in Fig. 8b. The pseudo-iR losses are caused by the uncompensated thin-layer diffusion within the porous structure of the TC and the PF electrodes^80^. The area-specific pseudo resistance (calculated from the slope of the linear ohmic region) was 4.57 times larger for the TC electrode (1.59 mΩ cm^2^) than that for the PF electrode (0.45 mΩ cm^2^). We attribute the lower pseudo-iR resistance exhibited by the PF electrode to the larger pore sizes which provided channels for improved mass transport (as evident from Sections 3.1.1, 3.1.2, and 3.2.1), and the higher wettability (as evident from Section 3.1.5).

Finally, losses due to concentration polarization in the TC electrode were apparent at a current density of 80 to 127 mA cm^−2^, and were significantly larger than that resembled by the PF electrode over the same current density region, providing important evidence that the presence of macro pores in the PF electrode played an important role in maintaining excellent mass transport properties, which are highly desirable for high current density and high power density operations of VRFBs.

#### Charge-Discharge test

**Figure 9:**
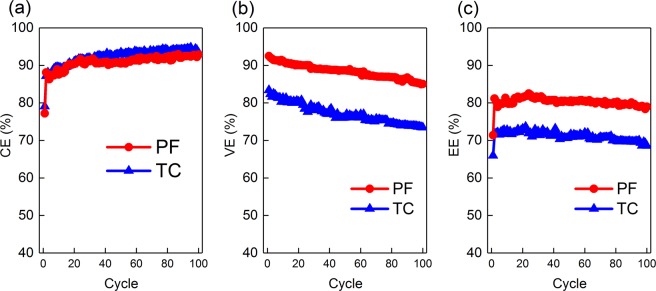
Efficiencies from charge discharge cycling results at a constant current density of 50 mA cm^-2^, showing the (**a**) coulombic efficiencies (CE), (**b**) voltage efficiencies (VE), and (**c**) energy efficiencies (EE) for the PF and the TC electrodes. The results show the higher VE and EE obtained by the PF electrodes as compared with the TC electrodes. The improvement in performance is attributed to the larger pore sizes of the PF electrodes.

Charge–discharge cycling was performed using a single layer of each of the fabricated electrodes in the positive and the negative half-cells utilizing an electrolyte of 1.7 M (VOSO_4_) in 3 M H_2_SO_4_ as the positive electrolyte and 1.7 M V^3+^ in 3 M H_2_SO_4_ as the negative electrolyte, and was performed at a constant current density of 50 mA cm^−2^ over 100 cycles. The calculated efficiencies (*η*) over cycle number are calculated as given in Fig. 9. The coulombic efficiencies (CE) averaged 92% for the TC electrode and 90% for the PF electrode throughout the cycling test. These excellent CE values were due to the effective electrolyte separation by the Nafion® 117 membrane preserving the amount of charge in each half-cell. On the other hand, the voltage efficiencies (VE) averaged at 88% for the PF electrode and 77% for the TC electrode. The high VE for both electrodes can be attributed to the highly interesting properties of the free-standing MWCNT sheets which provide a very large surface area and a large number of active sites for the electrochemical conversion reactions to occur. The VE for the PF was higher by 11% than that of the TC electrode, which is attributed to the lower polarization losses (as evident from Section 3.2.3) and is related to the improvement in mass transport of the active species within the bulk of the porous electrode, made possible by the larger pore sizes (as described in Sections 3.1.1 and 3.1.2).

The energy efficiencies (EE = CE × VE) averaged 80% for the PF electrode and 71% for the TC electrode throughout the cycling test. These excellent EE values were due to the excellent electrochemical activity of the MWCNT free standing porous electrodes facilitating a high surface area and a large number of active sites for the reactions to occur. As compared to the performance of different electrodes reported in literature^11^ (Table S5 in Supporting Information), our electrodes performance is interesting, and through further development, they may be competitive for the industry, noting that our results were obtained using only a single MWCNT sheet only, with minimal flow cell setup optimizations.

## Conclusions

Our in-lab fabricated freestanding MWCNT sheets resembled interesting properties for the redox flow battery industry. Our method was capable of fabricating MWCNT sheets with and without the presence of macro pores, in a scalable and low energy-consuming process. Unlike the Tape Casted (TC) electrode, the Puffy Fiber (PF) electrode consisted of macro pores within its pore size distribution. The macro-porous PF electrode showed a larger BET surface area (343 m^2^ g^−1^) and a higher porosity (0.81) as compared with the TC electrode (255 m^2^ g^−1^ and 0.71), resulting in its improved mass transport properties, lower polarization losses, and higher efficiencies, as was evident from our cyclic voltammetry, EIS, polarization curve, and charge-discharge tests analysis. The results demonstrate that the presence of macro pores in MWCNT free-standing sheets can facilitate excellent mass transport properties; thus, enabling higher current density and power density operations for vanadium redox flow batteries. Our proposed sheets are also interesting for various applications such as super-capacitors, electrochemical sensing technologies, and water treatment applications.

## Supplementary information

Supporting Information - Nanoscopic and Macro-Porous Carbon Nano-foam Electrodes with Improved Mass Transport for Vanadium Redox Flow Batteries

## Data Availability

The data generated during and/or analyzed during the current study are available from the corresponding author on reasonable request.
