# Supplementary material for: Nanoscopic and Macro-Porous Carbon Nano-foam Electrodes with Improved Mass Transport for Vanadium Redox Flow Batteries
Source: Sci Rep. 2019 Nov 27;9:17655. doi: 10.1038/s41598-019-53491-w (PMC6881356; doi:10.1038/s41598-019-53491-w)
Supplement: Supplementary file 1 — Supporting Information - Nanoscopic and Macro-Porous Carbon Nano-foam Electrodes with Improved Mass Transport for Vanadium Redox Flow Batteries [file 41598_2019_53491_MOESM1_ESM.docx]

**SUPPORTING INFORMATION**

**Nanoscopic and Macro-Porous Carbon Nano-foam Electrodes with Improved Mass Transport for Vanadium Redox Flow Batteries**

Ibrahim Mustafa^a^, Rahmat Susantyoko^b^, Chieh-Han Wu^c^, Fatima Ahmed^c^, Raed Hashaikeh^d^, Faisal Almarzooqi^a^, Saif Almheiri^b,^^[[1]](#footnote-1)^

^a^ Department of Chemical Engineering, Khalifa University of Science and Technology, Masdar Institute, Masdar City, P.O. Box 54224, Abu Dhabi, United Arab Emirates

^b^ Research & Development Center, Dubai Electricity and Water Authority (DEWA), Dubai, United Arab Emirates

^c^ Department of Mechanical Engineering, Khalifa University of Science and Technology, Masdar Institute, Masdar City, P.O. Box 54224, Abu Dhabi, United Arab Emirates

^d^ Engineering Division, New York University Abu Dhabi, Abu Dhabi, United Arab Emirates





Figure S 1. TEM image of a representative CNT strand, confirming that it is multi-walled and has a diameter in the range of 10 to 13 nm.


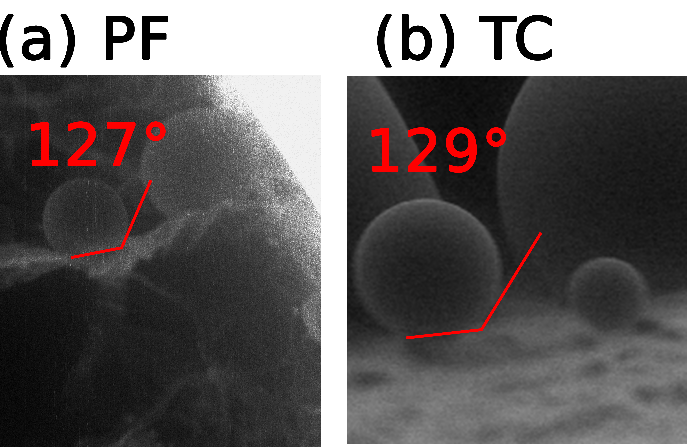


Figure S 2. Images for the (a) PF and the (b) TC electrodes showing similar contact angles for condensed droplets using environmental scanning electron microscopy.


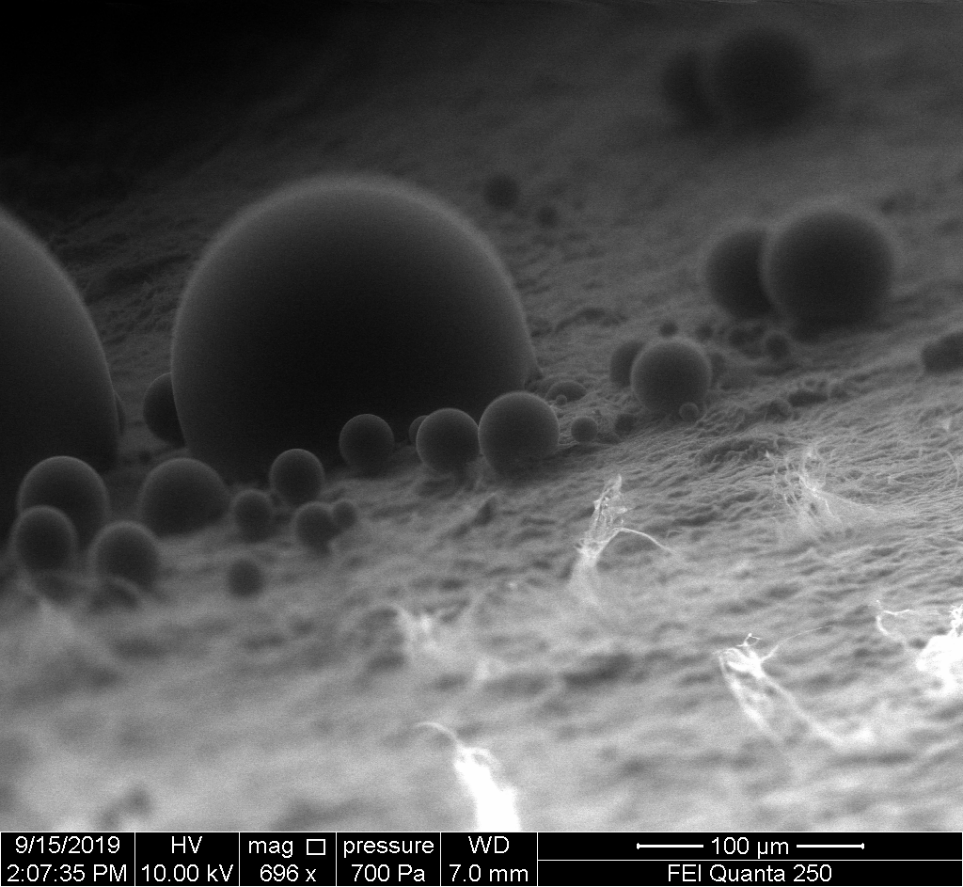


Figure S 3. Condensed water droplets formed on the surface of the TC electrode using E-SEM.





Figure S 4: Analysis of iR-corrected cyclic voltammograms for TC and PF electrodes at room temperature scanned for the negative and positive redox couples showing (a,b) plots of peak separations versus scan rate, (c,d) plots of peak ratios versus scan rate, and (e,f) plots of peak currents versus scan rate. The plots show the irreversibility of the electrodes. The TC electrode showed lower peak potential separations, while the PF electrode showed higher peak current densities.

Table S 1. Cyclic voltammetry data obtained in a solution of 0.05 M VO^2+^ in 3 M H_2_SO_4_ at various scan rates for the TC and the PF electrodes.^a^

| **Electrode** | $\boldsymbol{v}$ **(mV s^–1^)** | $\boldsymbol{j}_{\mathbf{pa}}$ **(mA cm^–2^)** | $\boldsymbol{j}_{\mathbf{pc}}$ **(mA cm^–2^)** | $\boldsymbol{E}_{\text{pa}}\boldsymbol{-}\boldsymbol{E}_{\text{pc}}$ **(mV)** | | ***I*_pa_/*I*_pc_** |
| --- | --- | --- | --- | --- | --- | --- |
| TC | 9 | 2.605 | -2.1536 | 43.34 | 1.21 | |
|  | 25 | 5.6922 | -4.6028 | 48.22 | 1.23 | |
|  | 49 | 9.913 | -7.169 | 67.74 | 1.38 | |
|  | 81 | 14.469 | -10.453 | 87.28 | 1.38 | |
|  | 100 | 17.027 | -11.398 | 94.6 | 1.49 | |
|  | 150 | 21.485 | -14.986 | 131.22 | 1.43 | |
|  | 200 | 27.2 | -17.572 | 153.24 | 1.54 | |
|  | 250 | 30.56 | -18.725 | 170.3 | 1.63 | |
| PF | 9 | 35.98 | -31.23 | 59.36 | 1.15 | |
|  | 25 | 77.51 | -61.77 | 95.98 | 1.25 | |
|  | 49 | 126.85 | -103.05 | 135.00 | 1.23 | |
|  | 81 | 175.26 | -144.10 | 179.01 | 1.21 | |
|  | 100 | 198.86 | -163.36 | 198.48 | 1.21 | |
|  | 150 | 262.14 | -198.23 | 235.17 | 1.32 | |
|  | 200 | 308.61 | -266.09 | 266.86 | 1.15 | |
|  | 250 | 376.33 | -280.76 | 298.65 | 1.34 | |

^a^ $v$ = scan rate; $j_{pa}$ = anodic peak current density; $j_{pc}$ = cathodic peak current density; $E_{\text{pa}}$ = anodic peak potential; $E_{\text{pc}}$ = cathodic peak potential; I_pa_ = anodic peak current; I_pc_ = cathodic peak current.

Table S 2. Cyclic voltammetry data obtained in a solution of 0.05 M V^3+^ in 3 M H_2_SO_4_ at various scan rates for the TC and the PF electrodes.^a^

| **Electrode** | $\boldsymbol{v}$ **(mV s^–1^)** | $\boldsymbol{j}_{\mathbf{pa}}$ **(mA cm^–2^)** | $\boldsymbol{j}_{\mathbf{pc}}$ **(mA cm^–2^)** | $\boldsymbol{E}_{\text{pa}}\boldsymbol{-}\boldsymbol{E}_{\text{pc}}$ **(mV)** | | ***I*_pa_/*I*_pc_** |
| --- | --- | --- | --- | --- | --- | --- |
| TC | 9 | 1.38 | -2.87 | 22.74 | 0.48 | |
|  | 25 | 5.19 | -9.50 | 49.59 | 0.54 | |
|  | 49 | 10.68 | -15.62 | 71.56 | 0.68 | |
|  | 81 | 17.15 | -22.14 | 93.54 | 0.77 | |
|  | 100 | 20.64 | -25.80 | 105.74 | 0.80 | |
|  | 150 | 24.16 | -33.22 | 125.28 | 0.72 | |
|  | 200 | 29.79 | -38.83 | 152.13 | 0.76 | |
|  | 250 | 34.92 | -45.46 | 171.66 | 0.76 | |
| PF | 9 | 17.15 | -41.37 | 69.28 | 0.41 | |
|  | 25 | 30.65 | -81.24 | 110.78 | 0.37 | |
|  | 49 | 50.09 | -122.54 | 142.52 | 0.40 | |
|  | 81 | 63.38 | -169.94 | 179.14 | 0.37 | |
|  | 100 | 80.65 | -209.26 | 198.66 | 0.38 | |
|  | 150 | 89.90 | -247.59 | 227.96 | 0.36 | |
|  | 200 | 122.67 | -296.29 | 252.38 | 0.41 | |
|  | 250 | 115.50 | -339.71 | 274.06 | 0.34 | |

^a^ $v$ = scan rate; $j_{pa}$ = anodic peak current density; $j_{pc}$ = cathodic peak current density; $E_{\text{pa}}$ = anodic peak potential; $E_{\text{pc}}$ = cathodic peak potential; I_pa_ = anodic peak current; I_pc_ = cathodic peak current.

**Diffusion Coefficients:**

Since both electrodes follow irreversible electrochemical behavior, and resemble a quasi-reversible response, the diffusion coefficient was calculated from the cyclic voltammograms at different scan rates, using the following relation:

$$I_{p}= \pm0.436 nFAC \sqrt{\frac{nFDv}{RT}}$$

Where $I_{p}$ is peak current, *n* is the number of electrons, *F* is the Faraday constant, A is the electrode area, *v* is the scan rate, *D* is the diffusion coefficient, R is the universal gas constant, and T is the temperature in kelvin.

Figure S 5 - Plots of cathodic and anodic peak currents vs. square root of scan rate, derived from plotted cyclic voltammograms.

The plots show linear fits with R^2^ values > 0.97, confirming the linear correlation between the peak currents and the square root of scan rate.

Table S 4 - Constant phase element (CPE) and double layer capacitance $\left( C_{\text{dl}} \right)$ values obtained by fitting the electrochemical impedance spectra.

| **Electrode** | **Electrolyte** | **CPE_A_** | **n** | $\boldsymbol{C}_{\text{dl}\text{\_A}}$  **(µF cm^–2^)** | **CPE_B_** | **n** | $\boldsymbol{C}_{\text{dl}\text{\_B}}$  **(µF cm^–2^)** |
| --- | --- | --- | --- | --- | --- | --- | --- |
| TC | Negative | 0.014989 | 0.85 | 5827.30 | 0.039226 | 0.91 | 37778.80 |
|  | Positive | 0.0087 | 0.99 | 8697.60 | N/A |  | N/A |
| PF | Negative | 0.035451 | 0.90 | 27471.60 | 0.15649 | 0.91 | 209148.00 |
|  | Positive | 0.01707 | 0.99 | 17067.00 | N/A |  | N/A |

Table S 5. Performance of different electrodes reported in literature from selected charge discharge experiments

| **Electrode** | **Cutoff Potentials (V)** | ***j***  **(mA cm^-2^)** | **Membrane** | **Electrolyte** | **Flow Rate**  **(mL min^-1^)** | **VE**  **(%)** | **EE**  **(%)** | **Ref.** |
| --- | --- | --- | --- | --- | --- | --- | --- | --- |
| Puffy Fiber (PF) | 1.7/1.1 | 50 | 117 | 90mL 1.7M V in 3M H_2_SO_4_ | 30 | 88 | 80 | This work |
| Tape Casted (TC) | 1.7/1.1 | 50 | 117 | 90mL 1.7M V in 3M H_2_SO_4_ | 30 | 77 | 71 | This work |
| Buckypaper 6x 5min (BP) | 1.7/ 0.8 | 75 | 115 | 15mL 1M V in 3M H_2_SO_4_ | 40 | 64.3 | 60 | ^46^ |
| Thermally activated GF (420°C for 10hrs) | 1.65/ 0.8 | 80 | 115 | 50mL 1.5M V in 2M H2SO4 | 60 | 87 | 80 | ^12^ |
| 10AA Carbon Paper | 1.7/1.1 | 80 | 115 | 20mL 1M V in 3M H_2_SO_4_ with Bi ions in the negative side | 180 | 90 | 85 | ^11^ |

Abbreviations: *j*, Applied current density; CE, coulombic efficiency; VE, voltage efficiency; EE, energy efficiency; BP, buckypaper; CP, carbon paper; GF, graphite felt.

1. Corresponding author. Tel.: +971 2 810 9995; Fax: +971 2 810 9901.

   E-mail addresses: contact@saifalmheiri.com (S. Almheiri). [↑](#footnote-ref-1)
